# Barriers to Optimal Tuberculosis Treatment Services at Community Health Centers: A Qualitative Study From a High Prevalent Tuberculosis Country

**DOI:** 10.3389/fphar.2022.857783

**Published:** 2022-03-25

**Authors:** Ivan S. Pradipta, Lusiana R. Idrus, Ari Probandari, Irma Melyani Puspitasari, Prayudi Santoso, Jan-Willem C. Alffenaar, Eelko Hak

**Affiliations:** ^1^ Department of Pharmacology and Clinical Pharmacy, Faculty of Pharmacy, Universitas Padjadjaran, Bandung, Indonesia; ^2^ Drug Utilization and Pharmacoepidemiology Research Group, Center of Excellence in Higher Education for Pharmaceutical Care Innovation, Universitas Padjadjaran, Bandung, Indonesia; ^3^ Unit of Pharmaco-Therapy, Pharmaco-Epidemiology and Pharmaco-Economics (PTE2), Groningen Research Institute of Pharmacy, University of Groningen, Groningen, Netherlands; ^4^ Bekasi General Hospital, West Java Local Government, Bekasi, Indonesia; ^5^ Department of Public Health, Faculty of Medicine, Universitas Sebelas Maret, Surakarta, Indonesia; ^6^ Disease Control Research Group, Faculty of Medicine, Universitas Sebelas Maret, Surakarta, Indonesia; ^7^ Division of Respirology and Critical Care Medicine, Department of Internal Medicine, Faculty of Medicine Universitas Padjadjaran, Dr. Hasan Sadikin General Hospital, Bandung, Indonesia; ^8^ Sydney Institute for Infectious Diseases, University of Sydney, Sydney, NSW, Australia; ^9^ Faculty of Medicine and Health, School of Pharmacy, University of Sydney, Sydney, NSW, Australia; ^10^ Werstmead Hospital, Sydney, NSW, Australia; ^11^ Department of Clinical Pharmacy and Pharmacology, University Medical Centrum Groningen, Groningen, Netherlands

**Keywords:** tuberculosis, Indonesia, tuberculosis barriers, healthcare problems, qualitative

## Abstract

**Background:** Community health centers (CHCs) are a backbone healthcare facility for tuberculosis (TB) services. Identifying barriers amongst TB service providers at the CHC level is required to help them deliver successful TB treatment.

**Aims**: The current study aimed to analyze barriers to successful TB treatment from the perspective of TB service providers at the CHC level in a high prevalent TB country.

**Methods:** A qualitative study was conducted using in-depth interviews and focus group discussions in a province of Indonesia with a high TB prevalence. Two districts representing rural and urban areas were selected to obtain information from TB service providers (i.e., physicians and nurses) at the CHC level. In addition, key informant interviews with TB patients, hospital TB specialists, pharmacists, and activists were conducted. The trustworthiness and credibility of the information were established using information saturation, participant validation, and triangulation approaches. The interviews were also transcribed for the inductive analysis using Atlas.ti 8.4 software.

**Results:** We identified 210 meaning units from 48 participants and classified them into two main themes: organizational capacity and TB program activities. We identified the inadequacy of human resources, facility, and external coordination as the main barriers to organizational capacity. Furthermore, the barriers were identified regarding TB program activities, that is, inadequate TB case finding, diagnosis, drug supply chain and dispensing management, treatment and monitoring, case recording and reporting, and public-private collaboration.

**Conclusion:** Strengthening CHCs in the management of TB is critical to reaching the national and global goals of TB eradication by 2035. These findings can be considered to develop evaluation strategies to improve the successful TB treatment in high prevalent TB countries, especially Indonesia.

## 1 Introduction

Tuberculosis (TB), a disease caused by *Mycobacterium tuberculosis* (M.tb), has been a continuous global threat (Al-Humadi et al., 2017; WHO, 2021). A recent global report has validated that an estimated 9.9 million people developed TB and that 1.3 million people died from TB in 2020 (WHO, 2021). Moreover, TB has been one of the top 10 causes of death and the leading cause of death from a single infectious agent worldwide (WHO, 2020). Drug-resistant-TB (DR-TB), a resistance of M.tb to one or more anti-tuberculosis drugs, is reported as the main challenge in TB treatment that significantly impacts the clinical and economic aspects of the patients (Pradipta et al., 2019a, 2019b; WHO, 2020; Byun et al., 2021). A global meta-analytical study from our group verified that TB patients previously treated for TB have a higher risk of developing multi-drug-resistant TB (MDR-TB) (Pradipta et al., 2018). Evidently, treatment barriers have occurred amongst these TB patients and potentially led to the DR-TB and unsuccessful TB treatment.

The updated global report showed that two-thirds of the new TB cases were developed in the eight high prevalent TB countries that mostly are in the lower-middle-income countries (WHO, 2021). A situational analysis was performed among ten high-burden countries: Bangladesh, China, India, Indonesia, Myanmar, Nigeria, Pakistan, Philippines, Russian Federation, and South Africa. The study showed that none of the countries is capable of providing effective care for 50% of the estimated drug-resistant TB patients (Monedero-Recuero et al., 2021). It underlines the need for intensified study in the high prevalent TB countries to provide a comprehensive picture of TB problems for the global strategies.

Globally, Indonesia is the third-ranked country regarding its contribution to developed TB worldwide (WHO, 2021). In 2019, the national TB case rate was estimated at 845,000 cases with a treatment coverage of approximately 66% and a total TB notification of nearly 567,000 cases (WHO, 2020). The situation worsened after the COVID-19 pandemic hit Indonesia. In 2020, TB treatment coverage decreased to a low 47%, and then TB case notification was reduced to roughly 393,000 cases (Ministry of Health Republic of Indonesia, 2021; WHO, 2021). Strengthening TB management is required to achieve global and national targets by 2035.

Community health centers (CHCs) have vital roles in managing tuberculosis in the community. The CHC is a frontline facility in managing TB cases in the high prevalent TB countries. In Indonesia, TB service providers at the CHC level have responsibilities to prevent, detect, diagnose, treat, monitor, notify, and report TB cases at the sub-district level (Ministry of Health Republic of Indonesia, 2010). They need adequate support from organizational and individual perspectives to perform qualified TB care in the community. In an earlier study from our group, several problems of TB care from the patient perspective were identified (Pradipta et al., 2021). The recent study highlighted potential problems from the perspective of TB service providers, such as TB stigma in a health facility, suboptimal treatment service, and negative perception of the quality of CHC facility (Pradipta et al., 2021). To the best of our knowledge, there is still limited study that analyzed problems in managing TB cases at the primary healthcare level from TB service providers’ perspective in high prevalent TB countries, including Indonesia. Describing the problems at community health centers in a high prevalent TB country can provide lessons learned for other TB high-burden countries that have similarities of the healthcare system and population characteristics. As such, insight is instrumental in developing effective and comprehensive strategies for improving TB care, and a qualitative study was conducted in Indonesia to explore barriers to treatment success from the perspective of TB service providers in CHCs.

## 2 Materials and Methods

We conducted a qualitative study using a phenomenological approach to provide a deep understanding of “what” was experienced and “how” several individuals experienced it (Creswell and Poth, 2007). The data were obtained employing the methodological beliefs as the philosophical assumption and the social constructivism as the interpretive framework (Creswell and Poth, 2007). Following those principles, the emergent idea was inductively obtained through methodological procedures, such as interviewing, observing, and analyzing the texts (Creswell and Poth, 2007). We obtained the information from the participant’s views of the situation regarding the study objective. The meanings were then formed through the interaction among the participants (social construction) rather than starting with a complex theory (Creswell and Poth, 2007).

Given the cultural and potential gender bias, two researchers with different gender were involved in the data collection. ISP (male) is a researcher who has followed training on quantitative and qualitative studies and has experience conducting public health studies involving in-depth interviews, focus group discussions (FGDs), and observational studies. LRI is a hospital pharmacist following a Ph.D. program that focuses on improving treatment outcomes on TB disease. There was no prior relationship between researchers and participants to minimize the potential imbalance of power that can affect the quality of the interviews and discussions. The participants were fully aware that all activities performed in this study aimed to improve TB healthcare services in Indonesia.

### 2.1 Context and Setting

**FIGURE 1:**
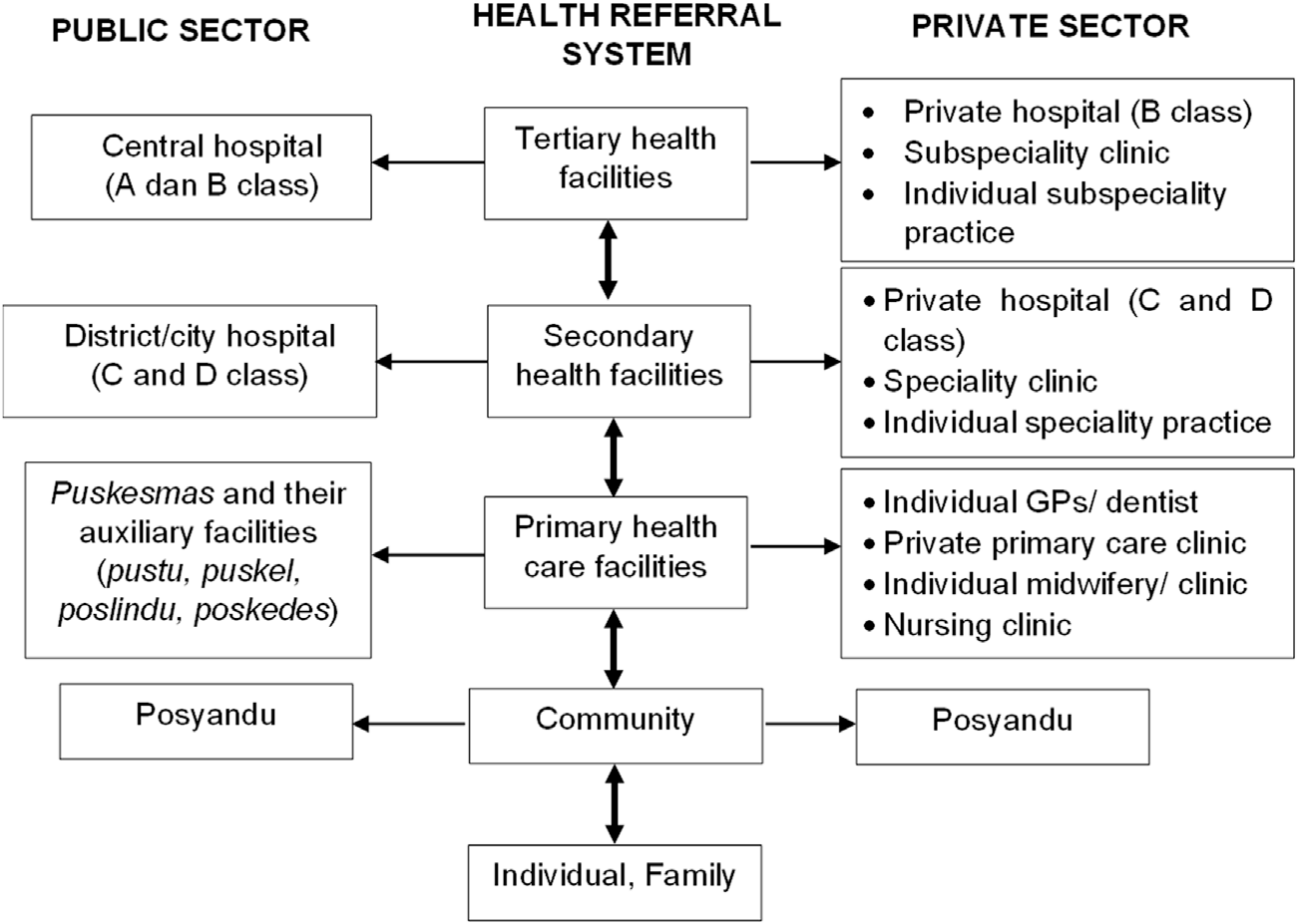
Indonesia health referral system. Volunteers (cadres) operate posyandu for vulnerable population groups at the community level in their area supervised by puskesmas*.*

The current study was conducted in a province of Indonesia that has a high TB prevalence nationally (Ministry of Health Republic of Indonesia, 2018). Two districts were selected as the research object representing rural and urban areas considering the different characteristics of the public health facility. Generally, CHC is a public health facility established at the sub-district level managed by the local government in Indonesia (Claramita et al., 2017). Given the catchment area and the high population at the sub-district level, several CHCs were supported by their auxiliary facilities (pustu, puskel, polindes, and poskesdes). The healthcare system in Indonesia is divided into public and private healthcare facilities. The governance of the public health system of Indonesia follows a gradual referral system covered by national health insurance for health services. Figure 1 describes the Indonesia health referral system modified from Claramita et al. (2017).

In Indonesia, TB units exist in each CHC, and staff members operate the unit. They have responsibilities to prevent, detect, diagnose, treat, monitor, notify and report TB cases at the sub-district level. The number of TB service providers at the CHC level depends on the availability of the healthcare staff members. Commonly, it comprises two to four healthcare personnel who have a medical doctor, nurse, and/or lab analyst background. In the meantime, the staff responsible for TB service should also operate another healthcare or health program at the CHC level. Referral hospitals provide care to support TB care at the CHC level concerning MDR-TB management. Nonetheless, not all referral hospitals at the district level have MDR-TB care. MDR-TB is managed in several centralized hospitals commonly established in a tertiary hospital.

### 2.2 Sampling Strategy

We applied criterion sampling (Palinkas et al., 2015) to recruit the participants in this study, and we selected five CHCs in each district with high TB cases. Additionally, a TB service provider at CHC was considered eligible for inclusion if having at least 6 months of experience managing TB cases. Data saturation was utilized to determine the final number of participants during the interview process. We defined data saturation as no emergence of new information relevant to the study objectives.

### 2.3 Ethical Issues

We followed the principle of the Declaration of Helsinki in conducting our study. The study protocol was approved by the ethics committee of the Universitas Padjadjaran, Indonesia (no. 333/UN.6/Kep/EC/2019). All the participants were provided informed consent to participate in this study. Deidentified participant and data protection were also applied during the study analysis to provide the confidentiality of participant information. The data were stored in a computer with password protection that can only be accessed by the principal researcher (ISP).

### 2.4 Data Collection

The participants were selected using gatekeepers from the district health offices (Hennink et al., 2015). We attempted to maximize the characteristics of the participants considering several characteristics: the distance level of CHC to the central district, existing TB case managed, age, and experiences in TB management. We then contacted the TB service providers to conduct FGDs. Prior to the focus groups, the written informed consent forms were delivered to the eligible participants at least a week before the FGD, providing sufficient time to decide on participation in this study. Furthermore, we conducted an FGD with a maximum of five participants per group. Considering the culture and level of authority, we separated the FGD on the basis of their backgrounds (physicians and paramedical staff) to have factual information from the participants. To validate the information obtained, we also attempted to collect information using structured interviews from the other participants, such as the TB coordinator at the district level, health district office staff, TB patients, or other relevant subjects, based on the information from the FGDs. The in-depth interviews (IDIs) were applied for the participants who potentially shared sensitive issues and were favored at a specific time due to time availability. The data were collected from February to April 2019.

Each interview started with general questions using Bahasa Indonesia, and then the interviewer explored the information based on pre-established research questions. The general questions were “what are your activities in TB management?” and “what and how are the TB treatment problems?” The interview followed several steps according to the interview guide shown in Supplementary File S1.

### 2.5 Data Processing and Analysis

All the participant information was recorded using audio for IDI and audio-visual for FGD. The recordings were transcribed and sent to the participants for member checking and approval. Thereafter, the approved transcripts were anonymously transferred to the Atlas.ti 8 software for data analysis.

The inductive data analysis was performed. It generally followed several steps, including familiarization, thematic framework identification, codification, and interpretation (Hennink et al., 2015). The familiarization of content aimed to construct a thematic framework. Once the general thematic framework was created, coding was performed by identifying the emergent meaning unit from the transcript and field notes. According to the created general thematic, the codes were classified into themes. Then, the sub-themes were developed by identifying the pattern of shared meaning across the codes, which can support the understanding of a phenomenon and were relevant to the study objective.

Moreover, data interpretation was performed by analyzing the code pattern amongst the participants. Potential relationships across the codes were also investigated through co-occurring codes, which overlapped in a meaning unit. ISP coded the transcript data, classified them into themes/sub-themes, and then discussed them with LRI. The other researchers (AP, IMP, PS, JWA, and EH) reviewed the final concept’s codes, themes, and sub-themes. Any disagreements in the data analysis were resolved by the consensus considering the transcript and field notes.

### 2.6 Trustworthiness and Information Credibility

We combined data and investigator triangulation to enhance the trustworthiness of the information obtained from the participants. We observed the daily activities, facilities, and documents related to the findings in the data triangulation. The essential information from a participant was also confirmed to another participant based on the information context to ensure the information’s credibility. Investigator triangulation was performed by confirming the finding across the investigators without prior discussion. Lastly, we provided the member checking where the participants were allowed to read and approve the interview transcript without any pressure and guidance to agree with specific meanings to validate the interview, preserve research ethics, and empower the participants related to the findings (Mero-Jaffe, 2011). The Standard for Reporting Qualitative Research (SRQR) was followed for a systematic and transparent report (O’Brien et al., 2014).

## 3 Results

The study successfully involved 18 of 20 eligible TB service providers at the CHC level for the FGDs and IDIs. Two TB service providers did not participate in the FGD due to the lack of backfill for daily clinical work in their CHCs. We conducted FGDs and IDIs with other relevant participants to validate and enrich information obtained from the 18 participating TB service providers. The other group comprised TB programmers at the district health office level, pharmacists at the CHC level, TB patients, a patient’s family member, TB programmers at the hospital level, and a representative of the pharmaceutical service department at the district health office. Thus, 48 participants distributed across rural (23 participants) and urban (25 participants) areas were included in this study. The average participant’s age was 40 years old (minimum = 16; maximum = 56), whilst the average participant’s experience in TB was 84 months (min = 6; max = 348). We performed six FGDs amongst 28 participants, that is, TB service providers (physician and nurse) and pharmacy staff at the CHC level, whilst the IDIs were performed amongst 20 participants. The average duration of the FGDs was 95.83 min (min = 69; max = 124), whilst the average duration of the IDIs was 37.80 min (min = 4; max = 117). Due to a hearing problem from a TB patient participant, an interview was ended at the minimum duration of the interview (4 min). We then continued the interview with his wife to explore the information needed. We interviewed the participants in several locations, that is, district health office (20 participants), community health service (21 participants), hospital (5 participants), participant’s home (1 participant), and non-government organization office (1 participant). Table 1 presents the participant characteristics.

**TABLE 1:** Characteristics of the participants (*n* = 48).

Characteristics	Male (*n* = 8)		Female (*n* = 40)	
	Rural (*n* = 4)	Urban (*n* = 4)	Rural (*n* = 19)	Urban (*n* = 21)
Tuberculosis service providers				
Physician at CHC level	1	0	4	3
Nurse at CHC level	0	0	5	5
TB service provider at DOH level	0	0	1	2
Pharmacist of the CHC	0	0	5	5
Healthcare workers at the hospital setting				
TB nurse	0	0	0	1
Pharmacist	0	0	0	1
Pulmonologist	0	0	0	1
Internist	0	1	0	0
Other supporting department staff				
Department of Pharmaceutical Services at DOH	1	0	0	1
Tuberculosis activist from a TB NGO	0	0	0	1
TB patients and their family members				
Non-MDR-TB patient	1	2	2	0
MDR-TB patient	1	1	1	1
Family members	0	0	1	0

Information: CHC: community health center; DOH: district health office; TB: tuberculosis; MDR-TB: multidrug-resistant tuberculosis; NGO: non-governmental organization.

**FIGURE 2:**
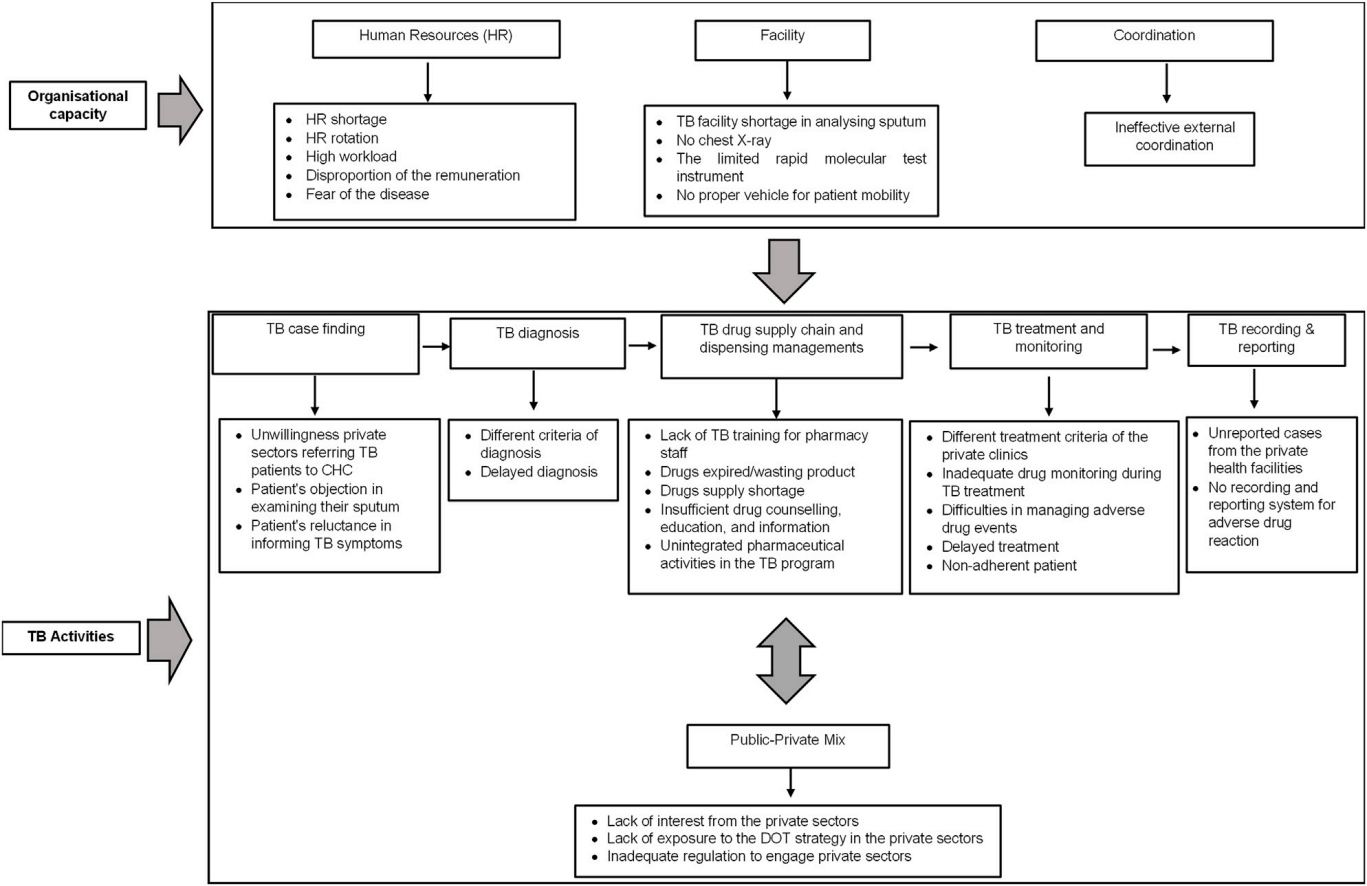
Theme, sub-theme, and code of barriers to successful TB treatment from the TB service provider perspectives.

We identified 210 meaning units that related to the study objective. The meaning units were inductively coded and classified into two major themes: organizational capacity and TB activities. The organizational capacity consists of three sub-themes: human resources (HR), facility, and coordination. The TB activities theme comprises six sub-themes: TB case finding, diagnosis, drug supply chain and dispensing management, treatment, recording and reporting, and public–private mix (PPM) activities. Figure 2 depicts the themes, sub-themes, and codes.

### 3.1 Organizational Capacity Theme

A total of 69 meaning units were analyzed for the organizational capacity theme. The organizational capacity barriers were related to HR (i.e., the HR shortage, high workload, disproportion of remuneration score system, HC fear, and HR rotation), facility (i.e., limited availability of sputum tests, chest X-rays, rapid molecular tests, and ambulances), and coordination (i.e., ineffective external coordination). More detailed information on the organizational barriers is described below.

### 3.1.1 HR

Our participants stated that the shortage of HR had been the main problem in managing TB. A TB analyst who operates a sputum test is not always available in every CHC:

“*We are confused to examine the sputum because we do not have a TB analyst in our laboratory” (CHC’s doctor 1).*

Limited healthcare providers also impacted TB treatment care. Our TB patient’s participant highlighted that he should find another healthcare facility to inject his medicine taken from the CHC due to the limited staff who operate TB care:

*“Because the TB staff does not provide TB care every day [*sic*]. They have activities in another programme of CHC, e.g., Posyandu or others, I should inject my TB medicines outside the CHC” (TB patient 1).*

The HR shortage implied the high workload of the TB service provider. It was worsened when TB service providers also confirmed their fears. Our participants stated that they should manage multiple activities other than TB care:

*“If there is a TB patient, other staff seem not to face the patient. They are worried about getting the infection” (CHC’s programmer/nurse 3).* and *“I have multiple jobs in CHC, so my job is not only taking care of TB but also other activities, e.g., emergency unit and another task” (CHC’s programmer nurse 1).*

Our participants mentioned that a higher remuneration should be applied amongst healthcare service providers who manage a risky disease. Given that TB pathogen can potentially infect them and spread the disease to their family, they thought that TB is a higher risk service than other services at the CHC level. Thus, the amount of remuneration should be adjusted on the basis of the potential risks:

*“We do not have a special consideration for the incentive to do the risky work. All programmes get the same remuneration point” (CHC’s programmer/nurse 2).*

The participants also disclosed that HR rotation for a skilled TB service provider at the district level is not under their control. It will take more effort to provide individual training when the new staff members do not have experience in TB management:

*“It makes me in the difficult situation when there is TB staffs’ rotation which already had TB knowledge and skills” (district TB programmer 1).*

### 3.1.2 Facility

The supported facility for TB activities in CHC is also highlighted. Our participants expressed limited facilities for TB diagnosis and transportation:

*“The problem here is we do not have a sputum corner for taking the patient’s sputum. Ideally, to get fast action, the patient can be asked to provide the sputum in our facility” (CHC’s doctor 2).*

Our participant stated that no special room could be used for collecting patient sputum at the CHC. The suspected TB patients were asked to provide the sputum specimen from their homes. However, in many cases, the patient did not send the sputum after being asked to send it, or they provided unqualified sputum for the test because of the inability to expel the sputum:

*“We face difficulty diagnosing TB patients because we only have AFB tests. The positive result from the AFB test is still rare to be found, so we have to refer suspected TB patients to have chest x-ray in the hospital. The problem is not every patient has free access to chest x-ray in the hospital” (CHC’s doctor 2).*

As described in the quotations above, our participant stated difficulties diagnosing TB due to no existing chest x-ray instrument at the CHC level. In some cases, suspected TB patients should be referred to the hospital for a chest X-ray examination because there were doubts about the result of the sputum test through the AFB test. However, not all suspected TB patients can access free chest x-ray examinations in the hospital due to not having healthcare insurance:

*“According to the national regulation, we should use rapid molecular test instrument. However, we only have one instrument covering around 42 CHCs in our district. You can imagine if all the CHC send the TB specimens, it will cause overcapacity and delay the result” (district TB programmer 1).*

Our participants stated that rapid molecular test (RMT) was the essential examination as it was recommended to diagnose TB according to the national guideline. However, there was only an RMT instrument that should cover 42 CHCs. It led to the delayed result of the test:

*“We have a cooperation with sub-district staff to borrow an office car for TB patients because we should accompany TB patients for MDR-TB treatment at the MDR-TB centre hospital. However, that is, *[sic]* only a usual car, that, is not an ambulance, so the personal safety is poor” (CHC’s programmer/nurse 3).*

Our participant described an experience when she accompanied an MDR-TB patient to an MDR-TB center for TB examinations without proper personal safety. No ambulance can be used, and finally, she used a usual car with poor personal safety for around 3 h trip.

### 3.1.3 Coordination

Our participants mentioned ineffective external coordination with the private healthcare (PRHC) facilities because of the different criteria of the diagnosis and treatment found. The participants communicated that they expressed difficulties coordinating with TB stakeholders in managing TB cases based on the national guidelines. They suggested providing advocacy to improve the coordination system in managing TB cases with the PRHC:

*“It is better to have an advocacy to all health care staff around the CHCs, medical doctors, nurses, midwives, and pharmacists. All are invited to explain that the management of TB cases should be uniform” (CHC’s programmer/nurse 4).*

### 3.2 TB Program Activity Theme

A total of 141 meaning units were analyzed in the TB program activity theme. We identified the TB activity barriers related to TB case finding (i.e., referral activity, patient’s objection in the sputum examination and patient’s openness); diagnosis (i.e., different criteria and delayed diagnosis); drug supply chain and dispensing management (i.e., lack of TB training, expired drug/wasting product, drug supply, insufficient drug counseling, education and information, and unintegrated pharmaceutical activities); treatment and monitoring (i.e., different treatment criteria, inadequate treatment monitoring, difficulties in managing adverse drug reaction, delayed treatment, and patient non-adherence); reporting (i.e., unreported case from the private sectors and no recording for adverse drug reaction); and PPM (i.e., lack of interest from private sectors, lack of Directly Observed Therapy (DOT) exposure, and inadequate local regulation). We provided below a detailed description of the barriers in the TB activity theme.

### 3.2.1 Tuberculosis Case Finding

We identified two barriers related to the TB case finding. According to the Indonesian health system, TB care can also be provided by PRHC. Nevertheless, not all PRHC have the proper facility to diagnose TB disease. This can lead to TB misdiagnosis and inappropriate treatment. Thus, the failure of PRHC to refer or report their TB patient to CHC has been identified as case-finding and reporting problems:

*“Especially for the private clinics, I have asked them to cooperate with us for patients with TB symptoms. Please check the patient with the rapid molecular test in our facility. If the result has been released, we will send back the patient to them. We also inform to use [*sic*] the DOT strategy for the TB regimen, but unfortunately, they reject our request” (CHC’s programmer/nurse 5).*

Moreover, we analyzed that exploring information about TB symptoms from suspected TB patients has been an obstacle in detecting TB cases. The suspected TB patients were reluctant to share information about their symptoms, and the suspected TB patients were reluctant to perform free sputum tests in CHC:

*“The difficulty is when we try to explore information from the suspected TB patient. Are there coughing symptoms in your family? Then they did not want to open the information. They said that all are fine” (CHC’s TB programmer/nurse 6) and “In my case, we have identified ten suspected TB patients who should be tested for the sputum. We have given them the sputum pot, but, although it is free, [*sic*] mostly did not go to check the sputum” (CHC’s TB programmer/nurse 4).*

### 3.2.2 Tuberculosis Diagnosis

Our participants communicated several problems in the TB diagnosis activities: delayed diagnosis and distinct diagnosis criteria from outside health facilities. We found that the misdiagnosis in PRHC facilities caused the patients to experience delayed TB diagnosis:

*“We commonly find TB patients with delayed diagnosis. They just went to the health facility after they had the TB symptom too long or they already went to health facilities several times, but the diagnosis is not TB” (TB patients 2).*

An MDR-TB patient informed that his sputum was not examined when he had been diagnosed with TB. A chest radiograph was the only supported examination when he had a TB-sensitive diagnosis in a PRHC facility. However, the regimen was changed to an MDR-TB regimen at CHC when the sputum test showed that the pathogen had been resistant to rifampicin:

*“I did not check my sputum. I just had a chest X-ray procedure and received the TB treatment” (TB patients 1).*

### 3.2.3 Tuberculosis Drug Supply Chain and Dispensing

This study identified unintegrated pharmacy staff activities in the TB program. Moreover, TB training was not provided for pharmacy staff at the CHCs:

*“We have not provided tuberculosis training for pharmacy yet” (district TB programmer 2).*

Anti-TB drugs were stored and dispensed in the TB room rather than from the pharmacy, and the drugs were dispensed by a TB service provider (nurse) due to the easiness of providing the drugs for TB patients:

*“For drug storage, monitoring and information are conducted by TB programmer. The pharmacist has not performed those activities. The TB flow service is different. TB patients go to the back room directly, so they go to the TB service room operated by TB programmer directly” (CHC’s pharmacist 1).*

TB service providers insufficiently delivered drug counseling, education, and information. The limited time of TB service providers was due to other responsibilities in the CHC:

*“Patient education can take around 20–25 minutes. The problem is when we have a meeting or other patient care, we cannot do that” (CHC’s doctor 3).*

The study also revealed that expired anti-TB drugs and the shortage of the drug supply had been a concern. Those might be due to improper drug planning, procurement, and monitoring:

*“There were cases that the medicines exceeded the expired date. It might be too much procurement and inadequacy of drug monitoring” (pharmacist’s HDO 1) and “We already know that we should identify the patient who needs TB prophylaxis, but the problem is the dosage form of the medicine, Isoniazid 300 mg, is not available. All the drugs are in the fixed-dose combination forms” (CHC’s programmer/nurse 6).*

### 3.2.4 Tuberculosis Treatment and Monitoring

We identified five barriers in the TB treatment and monitoring activities: delayed treatment, different TB regimens, inadequate drug monitoring, non-adherence to medication, and difficulties in managing adverse drug reactions:

*“The patients sometimes come after they get critical conditions. They were not aware of the condition from the appeared symptom” (CHC’s TB programmer/nurse 7) and “[…] we sometimes receive TB patients who are treated in the outside with different regimens. We think why it is not the same with our guideline” (CHC’s doctor 4).*

As the described quotation above, our participants communicated that the delayed treatment was due to the unawareness of TB patients in identifying the signs and symptoms of TB. The patients went to the CHC when the condition had been severe. Regarding treatment regimens, in some cases, our participants stated that they found different TB regimens prescribed by another private clinic compared with the regimen of CHC. Therefore, it is more challenging to deliver appropriate drugs at CHCs:

*“Based on my observation, development of MDR-TB is commonly not caused by an inappropriate drug, but it is more the absence of patients during the treatment during the period”. CHC*’*s TB programmer/nurse 4.*

*“Some patients were undetected. They did not collect the medicine at CHC. We just knew when the medicine had piled up in the storage cabinet”. District TB programmer 2.*

*“We face problems on the management of adverse drug reaction. From 2015 until now, we have had difficulties managing patients with an adverse drug reaction. We should refer to a hospital, but the patient has been referred again to another hospital. It takes a long time to manage the patient”. CHC’s nurse 8.*

The other problems of medication were also highlighted in the quotation above. We identified that the development of MDR-TB was commonly due to the absence of TB patients in taking the medication. The patients did not come to CHC regularly to collect and take their TB medication. The problem was complex when the management of adverse drug reactions was not performed adequately at the CHC level. Our participants communicated that they face difficulties managing TB adverse drug reactions. Inability in managing TB adverse drug reactions at the CHC led the staff to transfer the patient to the hospital. However, the patients were not always ended in the first referred hospital. In some cases, the patients were referred again to another hospital, which can cause a delay in managing the incidence of TB adverse drug reaction.

### 3.2.5 Tuberculosis Recording and Reporting

We found that TB recording and reporting problems were related to unreported TB cases from the PRHC facilities and no proper recording and reporting system for the adverse drug reaction (ADR) of anti-tuberculosis drugs. Comprehensive TB case reporting is necessary to estimate TB burden and drug monitoring for successful treatment. As a public health facility, the CHC has to record and report all TB cases in their area. However, TB service providers stated that TB reporting from the PRHC facilities was difficult to collect:

*“If we want to report all cases, all the health facilities network must report their cases, including on-going and the completed cases. However, the problem is that we do not know the case when the patient has been treated in the private health facility” (CHC’s program/nurse 9).*

Furthermore, ADR recording and reporting were not performed optimally at the CHC level. It was confirmed by TB service providers that no ADR recording and reporting system is available in their CHCs:

*“Ideally, the adverse drug reaction monitoring form should be provided in CHC, but I have not reported it yet at my CHC” (CHC’s doctor 5).*

### 3.2.6 Public–Private Mix (PPM) Program

Given that public health facility holds considerable responsibilities in healthcare, collaboration with the private facilities under the PPM program is strongly recommended. Nonetheless, we observed that PPM at the CHC level faced several problems: lack of interest from PRHC, lack of exposure to the DOT strategy in the private sectors, and inadequate local regulation to engage PRHC. As described in organizational capacity, HR shortage and external coordination affected the performance of the PPM program at the CHC level:

*“We do not have any difficulties inviting local communities here, but it is complicated for the private health facilities”. CHC*’*s doctor 3.*

*“I think the problem here is the private doctor, hospital, or pharmacy have not been fully exposed to DOT strategy”. CHC*’*s doctor 6.*

*“We still do not have a strong regulation, such as a local regulation that explains reward and punishment for engaging private sectors under PPM TB program”. District TB programmer 2.*

## 4 Discussion

Our study demonstrated that organizational capacity is the main barrier affecting TB services at the CHC level. It includes adequate HRs, facilities, and effective external coordination with the relevant stakeholders. Further, barriers to daily clinical practice in managing TB cases at the CHC level were shown, such as inadequate TB case finding, diagnosis, drug supply chain and dispensing management, treatment and monitoring, recording and reporting, and PPM program. Notably, the PPM is essential for optimal TB care because it can associate with TB services at the CHC level.

We found that the absence of adequate staffing levels in the TB program is one of the main issues in managing TB cases at the CHC level. The limited healthcare staff generated a workload burden in the healthcare system in CHC, where they are obliged to provide health services for high numbers of patients per day, and the burden escalated by the additional works in another program or other healthcare activities. As TB is perceived as a high-risk transmission disease, TB service providers felt that the incentive to manage TB should differ from non-high-risk transmission diseases. Incentives could stimulate better performance and the long-lasting effects of the performance in healthcare (Abduljawad and Al-Assaf, 2011). Nevertheless, an incentive scheme should be developed. It can consider monetary and non-monetary incentives according to the financial and organizational conditions of CHC.

Our study demonstrated that replacing the experienced TB staff with the new staff without TB experience has been an obstacle to the continuity and success of the program. Given that the regular training program does not provide for the new staff, the daily TB care will be affected. We analyzed that the limited budget for providing the training and no comprehensive planning for the staff rotation at the local government level may be causal factors for the barriers regarding the staff rotation. It was in line with a survey that trained staff in TB was inadequate in many high prevalent TB countries (Figueroa-Munoz et al., 2005). Since the staff rotation at the CHC level in Indonesia under the local government authority, it should be realized by the local government that TB knowledge and skill deficit could lead to suboptimal TB care and infection control in health facilities (Shrestha et al., 2017). The problem was complex when HC fear was explained in this study because of potential TB transmission. Insufficient knowledge, skill, capability, and facility can be causal factors for HC fear that lead to TB-related stigma in health facilities (Chang and Cataldo, 2014; Nyblade et al., 2019; Probandari et al., 2019). Hence, ensuring the knowledge, skill, capability, and facility in managing TB cases is crucial in minimizing potential problems in TB care, including minimizing poor treatment outcomes and TB-related stigma from healthcare providers.

A shortage of TB facilities was identified in our study. Laboratory capacity to analyze sputum was limited, as well as the unavailability of several essential facilities, such as chest radiograph, rapid molecular test, and ambulance. According to the national guideline (Ministry of Health Republic of Indonesia, 2010), the sputum examination at the CHC level is critical to diagnosing TB. However, not all CHCs have a particular room for sputum collection, sufficient lab facility, and TB analysts. It leads to difficulties collecting and assessing qualified sputum from the suspected TB patients. Our observation and discussion with TB staff at the CHC found that limited laboratory staff and budget have played a vital role in providing particular sputum rooms at the CHC level. Consequently, in many cases, TB patients did not send the sputum to the CHC after being asked to provide the sputum at home, or they provided unqualified sputum for the test because of no guidance and inability to expel the sputum. An observational study in Indonesia supported our finding that a considerable number of TB suspects did not provide sputum in a proper quantity and quality (Sakundarno et al., 2009).

The unavailability of the chest X-ray is associated with the potential misdiagnosis of TB, delayed diagnosis, and treatment in our study. Our physician participant described that they complained about difficulties diagnosing TB without a chest X-ray, especially in the negative sputum smear patients. Additionally, the participant explained that the chest X-ray would be very beneficial because microscopic examination from the sputum of the patient does not always provide satisfactory results. However, although TB care is a mandatory service delivered by the CHC, the availability of chest X-rays at the CHC level is not supported by national regulation for the standard minimum instrument of TB care at the CHC (Ministry of Health Republic of Indonesia, 2019). The importance of chest X-ray for TB was studied in Indonesia. A cohort study showed that additional chest X-ray examination in the routine diagnostic workup for TB (i.e., clinical examination and sputum microscopy) provided high sensitivity and specificity in diagnosing TB in Indonesia (Saktiawati et al., 2019). This finding was also strengthened by a study that displayed the lack of confidence to diagnose TB amongst clinicians at the primary level, leading to delayed treatment (Lestari et al., 2020). Furthermore, we identified that delayed treatment is higher when the rapid molecular test to identify drug sensitivity does not always provide at the CHC level. The specimen should be referred to another CHC or health facility that takes several days. The government should notice the adequacy of the rapid molecular test in quality and quantity to overcome delayed diagnosis and treatment.

The unavailability of an ambulance for accompanying an MDR-TB patient for having an examination in the MDR-TB center was explained by our TB service provider participant. Given that the distance from CHC to the MDR-TB center is relatively far, she experienced accompanying an MDR-TB patient using a usual car without proper infection control during approximately 2 hours of the trip. Our study confirmed a previous qualitative study in the various settings of Indonesia that described the lack of facility for TB infection control in CHC (Probandari et al., 2019). The poor facility leads to having the unsafe feeling of TB service providers in managing TB patients (Probandari et al., 2019). It potentially discriminates or stigmatizes patients with TB in the health facility. A study in Mozambique exploring factors associated with poor quality TB care cascade underlines the importance of ensuring proper diagnostic facilities (Lisboa et al., 2020). The study concluded that ensuring the availability of diagnostic facilities will reduce delayed and inappropriate TB treatment (Lisboa et al., 2020).

As described by our participants, external coordination makes it difficult to engage the private sector in TB management. This factor can be associated with HR shortage to perform the partnership with the private sectors and insufficient local regulation to strongly engage the private sector in TB management. The regulation should declare the comprehensive PPM system, including the role, communication system, and incentive of all stakeholders related to PPM. We analyzed that the implementation of the PPM program is affected by the exposure of DOT strategies to the private sectors. Our study was supported by a case-control study in Bali, Indonesia. The study revealed that receiving information on DOT strategies is essential for engaging private clinics in managing TB cases (Putra et al., 2013).

In TB case finding and reporting, cooperation with the private sector is essential. Our study asserted that the unwillingness of private sectors to refer TB cases had been a problem in managing TB cases. Similarly, a study in Yogyakarta, Indonesia, has validated that almost one-third of the private practitioner participants never referred a TB suspect to a TB service provider (Mahendradhata et al., 2007). The need for cooperation with private sectors in Indonesia has been strengthened by the study that has confirmed that 73% of TB patients seek their first treatment in private sectors (Surya et al., 2017). Poor cooperation and coordination between the public and private sectors in managing TB cases will potentially lead to the under-reporting and loss of treatment follow-up TB cases. TB case-finding activities are more complex when TB suspects and their families are unaware of TB signs and symptoms. Improving public awareness on how they can identify TB and when and why they should visit a CHC may improve TB case detection.

### 4.1 Potential Limitations and Strengths

A limitation of this study is the lack of involvement from the private sector. The findings were analyzed from the perspective of the public sector’s participants. Thus, the additional information from private sector participants might enrich the findings. However, we believe that applying a collaborative study, defining proper criteria for the participant, performing triangulation and member checking, and following standard reporting for the transparent report in this study will contribute to the validity and reliability of the study.

### 4.2 Implication for Policy and Practice

Our study demonstrated that organizational capacity is essential in performing high-quality TB care at the CHC level. Given the CHC as a backbone facility in managing TB cases, this study has drawn several future directions that may be applied in the other high prevalent TB countries.

First, since the current national regulation (Ministry of Health Republic of Indonesia, 2019) has not covered the essential findings from this study as the standard minimum for TB care at the CHC level, there is a need to re-review and re-develop the minimum standard of human resources, facility, coordination system, and TB care at the CHC level. The development should consider the current evidence and challenge in improving treatment success and achieving national and global TB targets by 2035. The facilities that support TB care, such as sputum collection room, laboratory, rapid molecular test, and chest X-ray, should be adequately provided at the CHC level for rapid and precise TB diagnosis and treatment. Importantly, the quality and quantity of TB service provider personnel should be analyzed considering the workload, and TB analysts should be provided in each CHC. Second, a comprehensive organizational capacity assessment for the CHC is required nationally. The assessment will be beneficial in identifying the sub-standard care and developing its interventions in improving TB care at the CHC level (Cazabon et al., 2017). Third, the engagement of the private sector for TB care should be systematically performed by the CHC with sufficient guidance, facility, and support from the central and local government. The national guidelines and strategies should also be informed to PRHC facilities for similar concepts and principles in managing TB cases (Putra et al., 2013). Finally, strengthening pharmacy at the CHC in managing drug supply, dispensing, PPM program, and other patient-centered services (e.g., drug counseling and monitoring) can be initiated to improve TB treatment success at the CHC level (Abdulah et al., 2014; Miller and Goodman, 2020). The possibilities to implement digital health technology can be considered to support pharmacists in improving TB medication adherence and treatment outcomes (Ridho et al., 2022). Hence, systematic programs and guidance for the pharmacy staff should be developed to integrate pharmaceutical care in TB management at the CHC level.

In conclusion, as the central issues, several critical aspects in the organizational capacity are identified to improve the treatment success of TB patients: HR, facility, and external coordination. Those affect the activities in managing TB cases: TB case finding, diagnosis, drug supply and dispensing, drug monitoring, reporting, and recording. The willingness of the government to focus and resolve the mentioned issues is crucial to the continuity of the programmer and improving the successful TB treatment rates. Moreover, further studies are critical to quantify and generalize the finding to the population level to guide effective strategies and interventions to improve TB treatment success in high prevalent TB countries, especially Indonesia.

## Data Availability

The original contributions presented in the study are included in the article/Supplementary Material, further inquiries can be directed to the corresponding author.
